# Apparently-Different Clearance Rates from Cohort Studies of *Mycoplasma genitalium* Are Consistent after Accounting for Incidence of Infection, Recurrent Infection, and Study Design

**DOI:** 10.1371/journal.pone.0149087

**Published:** 2016-02-24

**Authors:** Timo Smieszek, Peter J. White

**Affiliations:** 1 NIHR Health Protection Research Unit in Modelling Methodology and MRC Centre for Outbreak Analysis and Modelling, Department of Infectious Disease Epidemiology, School of Public Health, Imperial College London, London W2 1PG, United Kingdom; 2 Modelling and Economics Unit, Centre for Infectious Disease Surveillance and Control, Public Health England, London NW9 5EQ, United Kingdom; University of Illinois at Urbana-Champaign, UNITED STATES

## Abstract

*Mycoplasma genitalium* is a potentially major cause of urethritis, cervicitis, pelvic inflammatory disease, infertility, and increased HIV risk. A better understanding of its natural history is crucial to informing control policy. Two extensive cohort studies (students in London, UK; Ugandan sex workers) suggest very different clearance rates; we aimed to understand the reasons and obtain improved estimates by making maximal use of the data from the studies. As *M*. *genitalium* is a sexually-transmitted infectious disease, we developed a model for time-to-event analysis that incorporates the processes of (re)infection and clearance, and fitted to data from the two cohort studies to estimate incidence and clearance rates under different scenarios of sexual partnership dynamics and study design (including sample handling and associated test sensitivity). In the London students, the estimated clearance rate is 0.80p.a. (mean duration 15 months), with incidence 1.31%-3.93%p.a. Without adjusting for study design, corresponding estimates from the Ugandan data are 3.44p.a. (mean duration 3.5 months) and 58%p.a. Apparent differences in clearance rates are probably mostly due to lower testing sensitivity in the Uganda study due to differences in sample handling, with 'true' clearance rates being similar, and adjusted incidence in Uganda being 28%p.a. Some differences are perhaps due to the sex workers having more-frequent antibiotic treatment, whilst reinfection within ongoing sexual partnerships might have caused some of the apparently-persistent infection in the London students. More information on partnership dynamics would inform more accurate estimates of natural-history parameters. Detailed studies in men are also required.

## Introduction

Sexually-transmitted *Mycoplasma genitalium* has recently gained increasing attention as a major cause of urethritis [1–3], cervicitis [1,2,4], pelvic inflammatory disease (PID) [1,2], infertility [2,5], and increased HIV risk [2,6]. Currently testing is not routinely done in, e.g., UK or USA, and there is only limited testing in Australia [7–10], so disease would be usually diagnosed syndromically, e.g., non-chlamydial non-gonococcal urethritis. Furthermore, frequent treatment failures [10–12] and high prevalence of macrolide-resistant strains [9,13–15] indicate suboptimal infection management and treatment [16,17].

*M*. *genitalium*’s impact on sexual and reproductive health necessitates a greater understanding of its natural history, including duration of untreated infection, proportion of infections that are symptomatic, and infectivity, to inform decisions regarding if and how public health interventions should be implemented. There have been calls for such interventions for *M*. *genitalium* [15–17], but we note that continued uncertainty regarding the natural history of *Chlamydia trachomatis* [18,19] means that the (cost-)effectiveness of screening remains unknown, and the interventions now in place complicate further study.

Two extensive cohort studies [20–23] have examined aspects of *M*. *genitalium*’s natural history in women, including rates of clearance of infection; intriguingly, the clearance rates were apparently very different. As cohort studies are difficult, expensive and time-consuming to conduct, it is important to obtain the maximum information from them.

In this paper we obtain refined parameter estimates through using an analytic approach that synthesizes additional evidence reported by the studies. In particular, we take into account that *M*. *genitalium* is a sexually-transmitted infection, and that members of the study cohort might acquire infection during the study, and that that those infected at enrolment might recover and become infected again by the time of follow-up, which would not be distinguishable from a persistent infection.

Furthermore, we aim to gain insight into the reasons for the differences reported by the studies, including potential reinfection within stable sexual partnerships (in a prolonged partnership if one partner is infected then there is an elevated risk that both partners will be infected, so that if one partner recovers during the partnership, there is an elevated risk of the person becoming infected again rapidly), to determine if the differences are likely to be due to differences in study design or due to ‘real’ biological or behavioral differences. Finally, we make recommendations for improving future studies to inform decisions regarding if and how public-health decision-making regarding if and how interventions should be implemented.

## Materials and Methods

We estimated the clearance rates from data that were collected in two cohort studies, one of students in London, UK, [20] and the other of Ugandan sex workers [21–23], using models that include acquisition of infection, including recurrent infection, as well as recovery. We further analyzed how two factors, (i) duration of sexual partnerships and (ii) test sensitivity, influence parameter estimates.

In this paper, we use “recurrent infection” to refer to an infection acquired after clearance of a previous infection, and “reinfection” as a recurrent infection that is acquired within a stable sexual partnership.

### Data

#### Female students, London, UK

**Fig 1:**
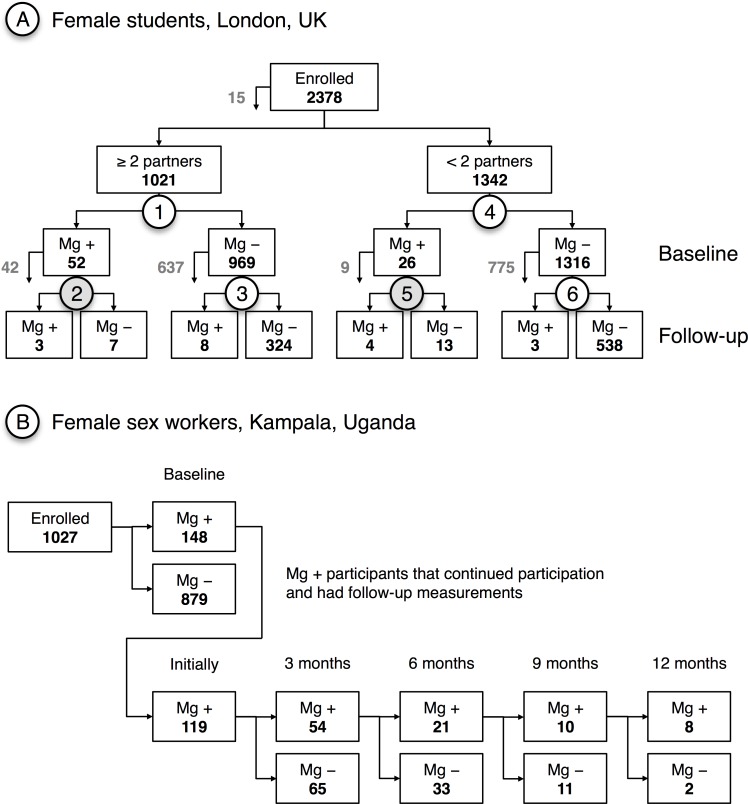
Flow charts of two cohort studies. (A) Oakeshott et al. [20]; numbers 1 to 6 are data items that are referred-to in the text; follow-up was after 12–21 months (median 16 months) for items 2 and 5 (grey), and after 11–32 months (median 16 months) for items 3 and 6; arrows exiting boxes indicate numbers of participants lost. (B) Vandepitte et al. [21]; numbers of *M*. *genitalium*-positive and -negative participants at baseline, as well as numbers of participants consistently *M*. *genitalium*-positive at consecutive time points, and numbers testing negative at each time point, who were not considered further in the analysis.

Oakeshott et al. [20] conducted a combined *C*. *trachomatis* and *M*. *genitalium* cohort study of female students in London (mean age: 21 years). Participants completed a sexual-behavior questionnaire at baseline and provided self-taken vaginal swabs at baseline and follow-up, 11–32 months later. Specimens were stored at -80°C until tested for *M*. *genitalium* by PCR. Fig 1A summarizes results.

#### Female sex workers, Kampala, Uganda

Vandepitte et al. [21–23] conducted a cohort study of women working in the sex industry in Kampala, Uganda (median age: 26 years [23]). Participants were asked to visit a project clinic at 3-month intervals, where they were repeatedly interviewed and where specimens were collected to be tested for HIV, HSV2, syphilis, gonorrhea, chlamydia, *T*. *vaginalis*, *Candida*, and *M*. *genitalium*. *M*. *genitalium* specimens were taken by project clinic staff using endocervical swabs. Those reporting STI symptoms or testing positive for any STI other than *M*. *genitalium* (which was tested for ex-post) were treated promptly. Specimens were brought to a laboratory within 12 hours and stored at -20°C until tested for *M*. *genitalium* by PCR. Fig 1B summarizes results.

More-detailed information about the study protocols is available in the related, freely accessible publications [20–23].

### Simulation model calibrated to the London-student data

**Fig 2:**
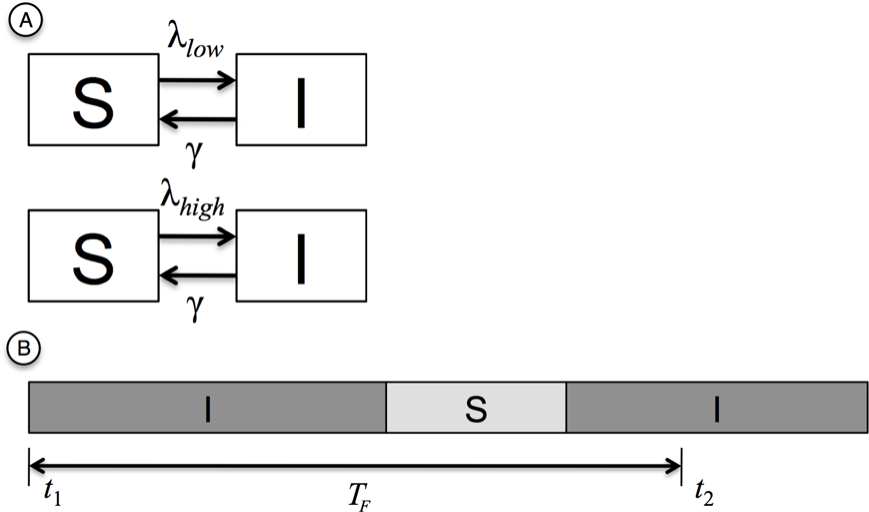
Schemata of model components. (A) Schema of London-student model. *S*: Susceptible state; *I*: Infected state; *λ*_*low*_: incidence rate of low-risk group; *λ*_*high*_: incidence rate of high-risk group; *γ*: recovery rate. (B) Schema of the succession of Infected and Susceptible states. *S*: Susceptible state; *I*: Infected state; *t*_1_: time at baseline; *t*_2_: time at follow-up; *T*_*F*_: time to follow-up; in this example, the individual was *Infected* at baseline and, again, at follow-up.

We represent individuals’ *M*. *genitalium* infection status with a ***S****usceptible*-***I****nfected*-***S****usceptible*-type model, in which individuals are in one of two discrete states: *Susceptible* (*S*, uninfected) and Infected (*I*), as shown in Fig 2A. These are the same states as used in a typical statistical survival (time-to-event) analysis; however, our model has the important difference that we also allow for individuals to become (re-)infected over time. We assume the prevalence of *M*. *genitalium*-positive individuals is stable over time and, hence, also the force of infection or incidence rate (*λ*) is stable.

The transition *S* → *I* occurs with rate *λ*_*low*_ for the London students with <2 partners in the previous year (low-risk group) and with rate *λ*_*high*_ for those with ≥2 partners (high-risk group), which are the groups reported by Oakeshott et al [20]. The transition *I* → *S* occurs with rate *γ* for all individuals. Hence, the infected period *T*_*I*_ has a mean of *γ*^-1^ and it is exponentially distributed with *exp*(*γ*). The susceptible periods *T*_*S*_ also follow exponential distributions with parameters *λ*_*low*_ and *λ*_*high*_, respectively. Individuals cycle between states *S* and *I*.

Our model aims at producing the overall best fit (maximum-likelihood) to six data items as in Fig 1A: the baseline prevalence in (1) the high-risk and (4) the low-risk group; the proportion of the initially infected individuals who were also infected at follow-up for (2) the high-risk and (5) the low-risk group; the proportion of those individuals whose initial *M*. *genitalium* test was negative that had a positive follow-up test for (3) the high-risk and (6) the low-risk group.

#### Prevalence

The probability *P*_I,i_ that any given individual *i* is *Infected* at any time *t*_*x*_ is given by *λ*/(*λ*+*γ*), with respective indices for low- and high-risk groups. Given the information about the prevalence in the low- and high-risk group (see Fig 1A), the log-likelihood functions are defined as

$$\text{ln}\;L_{prev,low}\left(\left[\begin{array}{cc} \lambda_{low} & \gamma \end{array}\right]|\mathrm{data}\right)=\sum_{i\in L_{+}}\text{ln}\;P_{I,i}\left(\lambda_{low},\gamma \right)+\sum_{j\in L_{-}}\text{ln}\;\left[1-P_{I,j}\left(\lambda_{low},\gamma \right)\right]$$ (1)

$$\text{ln}\;L_{prev,high}\left(\left[\begin{array}{cc} \lambda_{high} & \gamma \end{array}\right]|\mathrm{data}\right)=\sum_{k\in H_{+}}\text{ln}\;P_{I,k}\left(\lambda_{high},\gamma \right)+\sum_{l\in H_{-}}\text{ln}\;\left[1-P_{I,l}\left(\lambda_{high},\gamma \right)\right]$$ (2)

where *L*_+_ is the set of all study participants *i* who belonged to the low-risk group and tested positive for *M*. *genitalium* at baseline; *L*_−_ is the set of all study participants *j* who belonged to the low-risk group, but had a negative initial test result; *H*_+_ and *H*_−_ are the respective sets for the high-risk group.

#### Baseline versus follow-up measurements

The data in Fig 1A provide information about the infection status of all study participants at the baseline measurement, but also of many study participants at follow-up. The probability $P_{I(t_{2}),i}$ that individual *i* is *Infected* at time of follow-up, *t*_2_, depends on the infection status at baseline (i.e., at *t*_1_), the duration of the time period between baseline and follow-up measurement, *T*_*F*_, as well as on *λ*_*low*_, *λ*_*high*_ and *γ*.

Time to follow-up, *T*_*F*_, varied between study participants, and only the minimum, the maximum, and the median, *μ*_1/2_, are known for both the low- and the high-risk group (see Fig 1A). For any given individual *i*, *T*_*F*_ is within interval [min, *μ*_1/2_] with a probability of 50% and within interval [*μ*_1/2_, max] also with a probability of 50%. In absence of further information about the distribution, we assume that *T*_*F*_ follows a uniform distribution within each interval.

We determined the mean probability *P*_*I*_ that an individual is *Infected* at follow-up–given the infection status at baseline and whether the individual belongs to the high- or low-risk group–stochastically, by cycling through *Infected* and *Susceptible* states until *t*_2_ is passed (Fig 2B). The infection status at time *t*_2_ determines the infection status at follow-up. For each set of parameters, the estimate of *P*_*I*_ is based on at least 10^6^ realizations, and in areas of the parameter space with a high likelihood, we used up to 10^7^ realizations.

The likelihood functions *L*_*baseline+*,*low*_, *L*_*baseline+*,*high*_, *L*_*baseline–*,*low*_, and *L*_*baseline–*,*high*_ are defined analogous to Eqs 1 and 2, using the mean probability of being infected at *t*_2_ as described above.

#### Combined log-likelihood function and parameter space

The combined log-likelihood is defined as

$$\begin{array}{l} \text{ln}\;L\left(\left[\begin{array}{ccc} \lambda_{low} & \lambda_{high} & \gamma \end{array}\right]|\mathrm{data}\right)=\text{ln}\;L_{prev,low}\left(\left[\begin{array}{cc} \lambda_{low} & \gamma \end{array}\right]|\mathrm{data}\right)+\\ \;\text{ln}\;L_{prev,high}\left(\left[\begin{array}{cc} \lambda_{high} & \gamma \end{array}\right]|\mathrm{data}\right)+\\ \;\text{ln}\;L_{baseline+,low}\left(\left[\begin{array}{cc} \lambda_{low} & \gamma \end{array}\right]|\mathrm{data}\right)+\\ \;\text{ln}\;L_{baseline+,high}\left(\left[\begin{array}{cc} \lambda_{high} & \gamma \end{array}\right]|\mathrm{data}\right)+\\ \;\text{ln}\;L_{baseline-,low}\left(\left[\begin{array}{cc} \lambda_{low} & \gamma \end{array}\right]|\mathrm{data}\right)+\\ \;\text{ln}\;L_{baseline-,high}\left(\left[\begin{array}{cc} \lambda_{high} & \gamma \end{array}\right]|\mathrm{data}\right) \end{array}$$ (3)

We established the likelihood landscape for a parameter space defined by vector $\left[\begin{array}{ccc} \lambda_{low} & \lambda_{high} & \gamma \end{array}\right]$ for the intervals *λ*_*low*_ in [0.0050, 0.0500] with a step width of 10^−4^, *γ* in [0.01, 4.00] with a step width of 0.01, and *λ*_*high*_/*λ*_*low*_ in [2.5, 3.5] with a step width of 0.1.

The parameter combination with the highest overall log-likelihood value represents with maximal likelihood the ‘true’ recovery and incidence rates, given that the underlying model is a good representation of the ‘real’ infection and clearance processes. We refer to this parameter combination as maximum likelihood estimate (MLE).

#### Sensitivity analyses

Having incomplete information means that we have to make some assumptions, e.g., the exact distribution of the time to follow-up. We examined scenarios to analyse how two key assumptions affect our results.

**Time to follow-up:** We assume that the time lag between the baseline and the follow-up measurements is uniformly distributed between the minimum and median as well as between the median and the maximum. For the sensitivity analysis, we define two other, extreme scenarios: one scenario, where *T*_*F*_ = min with a probability of 50% and *T*_*F*_ = *μ*_1/2_ with probability 50%; and an alternative scenario, where *T*_*F*_ = *μ*_1/2_ or *T*_*F*_ = max, both with a probability of 50%.

**Partnership duration:** We do not have any information about partnership durations, and, hence, our model does not incorporate the effects of stable sexual partnerships on infection transmission dynamics. In monogamous, stable partnerships, the risk of acquiring infections is either considerably elevated above the population average (if the partner is infected) [24] or non-existent (if the partner is not infected), compared to a situation with frequent casual partnerships (and no stable partnership). To assess the potential bias introduced by our assumption, we analyze how our results would differ, if we were to assume that all individuals of the low-risk group with two consecutive positive tests were in a stable partnership and became reinfected within this partnership; in our modeling framework, these cases could not contribute to the estimation of the recovery rate and so are excluded in the sensitivity analysis.

### Model calibrated to Ugandan sex workers, including test sensitivity

In the study of sex workers in Uganda [21–23], sample-storage conditions and the type of specimens collected might have reduced sensitivity of testing relative to the London study [20], and we tested if this might be an explanation for the results having been different. We model both (i) the clearance of and recurrent infection with *M*. *genitalium* among the Ugandan sex-worker population–analogous to the model of the London students–and (ii) the detection of infection, allowing for false-negative test results. No information about differing levels of sexual activity are available and, hence, no further subdivision into risk groups is performed.

#### Prevalence

The observed prevalence from the participants’ first clinic visit is modeled–analogous to the London student study–as

$$\begin{array}{l} P_{I}=P_{sens}\cdot \frac{\lambda }{\lambda +\gamma }\\ \text{ln}\;P_{prev}\left(\left[\begin{array}{ccc} \lambda & \gamma & P_{sens} \end{array}\right]|\mathrm{data}\right)=\sum_{I}\text{ln}\;P_{I}\left(\lambda ,\gamma ,P_{sens}\right)+\sum_{S}\text{ln}\left[1-P_{I}\left(\lambda ,\gamma ,P_{sens}\right)\right] \end{array}$$ (4)

where *P*_*sens*_ denotes the test sensitivity and can take any value between 0 (i.e., the test does not detect any of the truly infected cases) and 1 (i.e., the test detects all infected cases and so produces no false-negative results). Set *I* includes all study participants who were infected as the first clinic visit, set *S* includes the complement.

#### Clearance

We model the expected distribution of the clinic visit at which the first negative test result occurred. A negative test result can be due to clearance (analogous to the London student model, see Fig 2B), or due to a false-negative test results. We model clearance and recurrent infection as for the London students, but record false-negative results with probability 1–*P*_*sens*_ for every clinic visit where the simulated individual is infected.

We repeated this procedure 5∙10^6^ times and obtained stochastic estimates for the probabilities *P*_2_, *P*_3_, *P*_4_ and *P*_5_ that any given individual had her first negative test result at the respective clinic visit, as well as for the probability *P*_*no*_ that an individual didn’t have a negative result throughout the entire duration of the study (i.e., consistently tested positive). This allows us to define a log-likelihood function for the clearance of *M*. *genitalium*:

$$\ln L_{clearance}\left(\left[\begin{array}{ccc} \lambda & \gamma & P_{sens} \end{array}\right]|\mathrm{data}\right)=\sum_{65}\ln P_{2}\left(\lambda ,\gamma ,P_{sens}\right)+\ldots +\sum_{8}\ln P_{no}\left(\lambda ,\gamma ,P_{sens}\right)$$ (5)

#### Consistency with London-student data

We tested if we could construct a model (overall log-likelihood functions was the sum of Eqs 4 and 5) using recovery rate estimates from the London-student data and which is consistent with the Ugandan sex-worker data (α level of 0.05) if we vary the relative testing sensitivity in the Ugandan study to obtain the best fit to the empirical data.

We tested consistency using the following recovery rates from the London-student data:

- *γ*, the maximum-likelihood estimate (MLE);
- *γ*_*UCL*_, the upper bound of the 95% confidence interval (CI) of the MLE;
- *γ*_*max*_, the highest possible recovery rate, for which the outcomes of the London-student model are not significantly different from the London-student data (*α* level of 0.05);
- *γ*_*stable*,*max*_, the highest possible recovery rate, when we assumed that all low-risk students, who were positive at baseline and follow-up, were in stable partnerships.

#### Recurrent infection in Ugandan sex workers

We further analyzed which of the parameter sets (consisting of incidence and recovery rate as well as sensitivity) are consistent with apparently-recurrent infections that were observed in the Ugandan sex workers: 39% of the women who seemed to have cleared infection (i.e. tested negative after previously testing positive) tested positive again within 3 to 6 months [21].

We used our model of the Ugandan sex workers to determine how likely a sequence of positive-negative-positive results in successive tests is for the 109 women who had a negative test result at the 2nd, 3rd, or 4th clinic visit. We then used a binomial test (two-sided) to determine if the parameter-model combination is consistent with the observed 39% recurrent infections.

### Confidence intervals and consistency

#### Confidence intervals for MLEs

We determined 95% confidence intervals for all MLEs with likelihood-ratio tests. The confidence interval is, hence, defined by

$$2\left[\text{ln}\;L\left(\hat{\theta }|\mathrm{data}\right)-\text{ln}\;L\left(\theta |\mathrm{data}\right)\right]\leq crit.$$ (6)

where $\hat{\theta }$ is the MLE, *θ* is any other parameter vector, and *crit*. is a critical value. Since the likelihood-ratio statistic follows approximately the *χ*^2^ distribution, we obtain the critical value for an *α*-level of 0.05 from this distribution.

#### Consistency between data and model

For all data items where the outcome was dichotomous (i.e., *Infected* versus *Susceptible*), we used a binomial test (two-sided) to determine if a specific parameter-model combination could possibly produce the observed data. If a parameter set produces results that are significantly different (*α*-level of 0.05) from any observed data item, we deem the respective parameter set as inconsistent with the data.

One data item from the Ugandan sex worker study–the clinic visit when the participant had her first negative test result, if at all–is represented as a contingency table. Statistical difference between the observed data and the expected model outcome was evaluated with the G-test (*α*-level of 0.05).

## Results

### Overview of results

**Fig 3:**
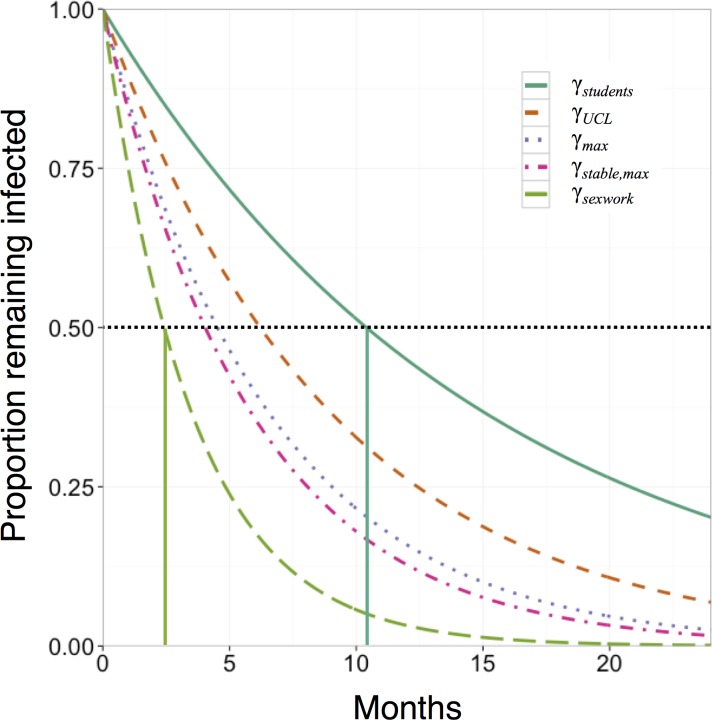
Duration of infection. Proportions of individuals remaining infected over time are shown for the various estimates of clearance rates, *γ*_*students*_ = 0.80p.a., *γ*_*UCL*_ = 1.34p.a., *γ*_*max*_ = 1.84p.a., *γ*_*stable*,*max*_ = 2.06p.a. (all based on the London-student data), and *γ*_*sexwork*_ = 3.44p.a. (based on the Ugandan sex-worker data); vertical lines mark time points when, on average, half of the initially infected individuals will have cleared the infection, depending on the respective recovery rate.

We present expected infection durations corresponding to the various estimates of clearance rate (from the two studies under different assumptions) in Fig 3, and describe the details of the analyses below.

### Parameter estimates for the London-student data

**Fig 4:**
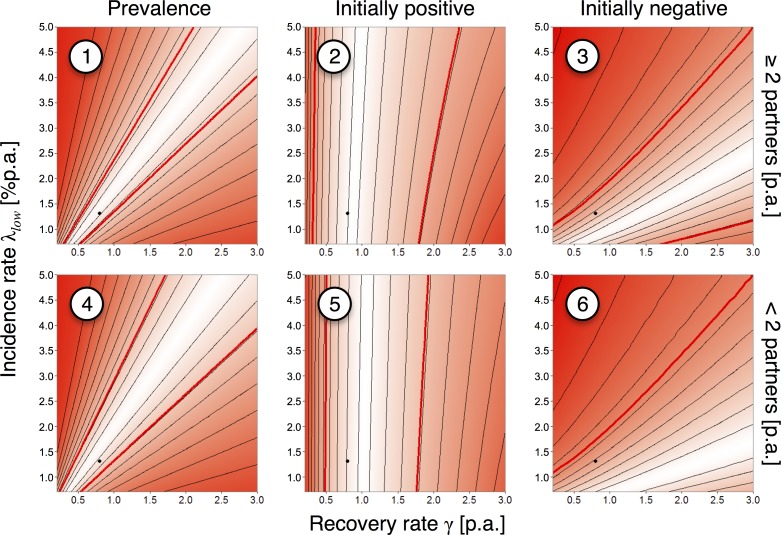
Likelihood landscapes by data item, London students. Upper and lower rows of the panels correspond, respectively, to the high-risk group (≥2 partners in the preceding year at baseline) and low-risk group (<2 partners); columns of the panels correspond to the prevalence at baseline, the proportion of positive tests at follow-up among the initially *M*. *genitalium*-positive participants, and the proportion of positive test at follow-up among the initially negative participants. Numbering corresponds to Fig 1A; black dots: overall maximum-likelihood estimate (MLE) for the recovery (*γ* = 0.80p.a.) and the incidence rates (*λ*_*low*_ = 1.31%p.a.); the ratio *λ*_*high*_/*λ*_*low*_ was fixed at the MLE of 3.0; thin, black contours: equal likelihood; colored areas: white indicates maximum likelihood, red indicates low likelihood; thick, red contours: parameter area where the model is consistent with the empirical data (*α*<0.05).

**Fig 5:**
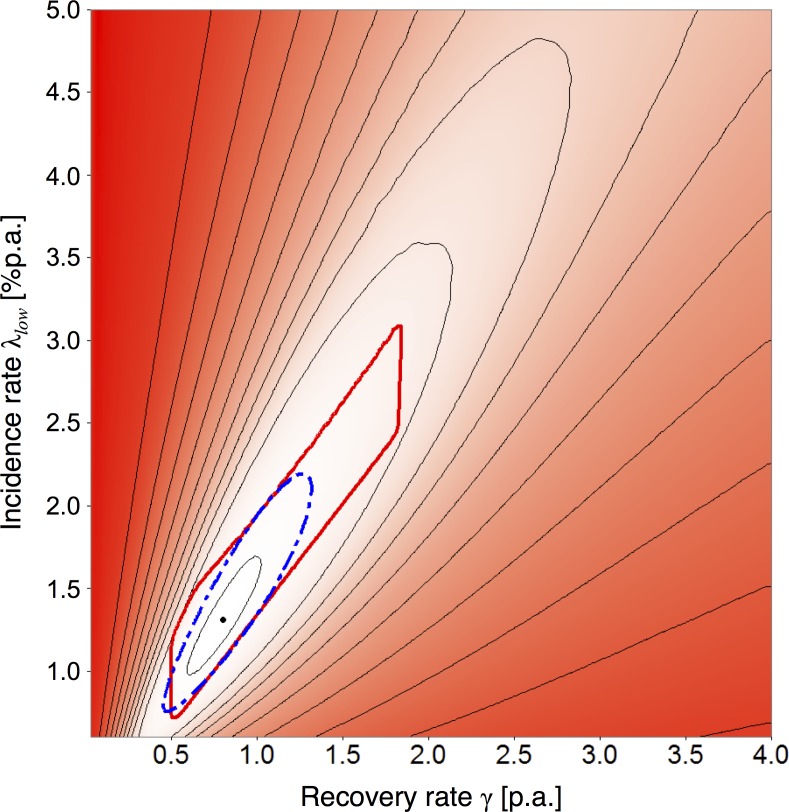
Recovery rate vs. incidence rate, maximum-likelihood estimate and plausible parameter range, London students. Black dot: maximum-likelihood estimate (MLE) for the recovery rate (*γ* = 0.80p.a.) and the incidence rate (*λ*_*low*_ = 1.31%p.a.); the ratio *λ*_*high*_/*λ*_*low*_ was fixed at the MLE of 3.0; thin, black contours: equal likelihood; combined likelihood for all six data items (see Fig 4); colored areas: white indicates maximum likelihood, red indicates low likelihood; thick, dashed, blue contour: 95% CI of the MLE; thick, red contour: parameter area where the model is consistent with the empirical data (*α*<0.05).

The best-fitting parameter combination, using all the available data items, is a recovery rate (*γ*) of 0.80p.a. (95% CI: 0.45–1.34; mean duration of infection of 15 months), with corresponding incidence rates, *λ*_*low*_ = 1.31%p.a. (95% CI: 0.75%-2.25%) and *λ*_*high*_ = 3.93%p.a. (95% CI: 2.25%-6.75%), since the most likely *λ*_*high*_/*λ*_*low*_ is 3.0 (Figs 4 and 5). For comparison, Oakeshott et al.’s [20] estimated incidence was 0.9% (95% CI: 0.5%-1.6%). However, a very wide range of parameter values are compatible with (i.e., not significantly different from) the empirical data (area within red contour lines, Fig 5). The maximal recovery rate (*γ*_*max*_) for which the model outcomes are not significantly different from the empirical data is 1.84p.a. (mean duration ~6.5 months).

Fig 4 shows the likelihood landscape for all six data items from Fig 1A separately. The parameters *λ* and *γ* are tightly linked in the cases of prevalence (1 and 4) and infection of initially uninfected participants (3 and 6): high *λ* requires high *γ* to reproduce the data; low *λ* requires low *γ*. The slope of the most likely parameter range is steeper for items 1 and 4 than for items 3 and 6. The MLE for *γ* is predominantly driven by the data on the number of initially *M*. *genitalium*-positive participants who were still positive at the time of the follow-up (items 2 and 5).

The estimates are rather insensitive to changes in the assumed distribution of times to follow-up. In the scenario using the longest times that were consistent with the reported minimum, maximum, and median yields *γ*_*h*_ = 0.75p.a. (95% CI: 0.44–1.23) and *λ*_*h*,*low*_ = 1.24%p.a. (95% CI: 0.74%-2.10%). In the scenario using the shortest times, *γ*_*l*_ = 0.86p.a. (95% CI: 0.51–1.5) and *λ*_*l*,*low*_ = 1.46%p.a. (95% CI: 0.86%-2.52%).

If we assume that all low-risk individuals who were positive both at baseline and follow-up were potentially reinfected within a stable partnership, and therefore removed from the analysis, then the best estimate for the recovery rate shifts to *γ*_*stable*_ = 1.34p.a. (95% CI: 0.66–2.18) with *λ*_*stable*,*low*_ = 2.17%p.a. (95% CI: 1.08%-3.00%), but the highest possible recovery rate that was consistent with all six data items was only *γ*_*stable*,*max*_ = 2.06p.a., which corresponds to a mean duration of infection of ~5.8 months.

### Clearance of *M*. *genitalium* infection in Ugandan sex workers

Clearance of *M*. *genitalium* in the Ugandan sex-worker cohort appeared to be relatively fast, compared with to the London-student cohort: a simple analysis, fitting an exponential curve to the five data points suggests a recovery rate *γ* = 3.14p.a., corresponding to a mean duration of infection of ~3.8 months.

**Fig 6:**
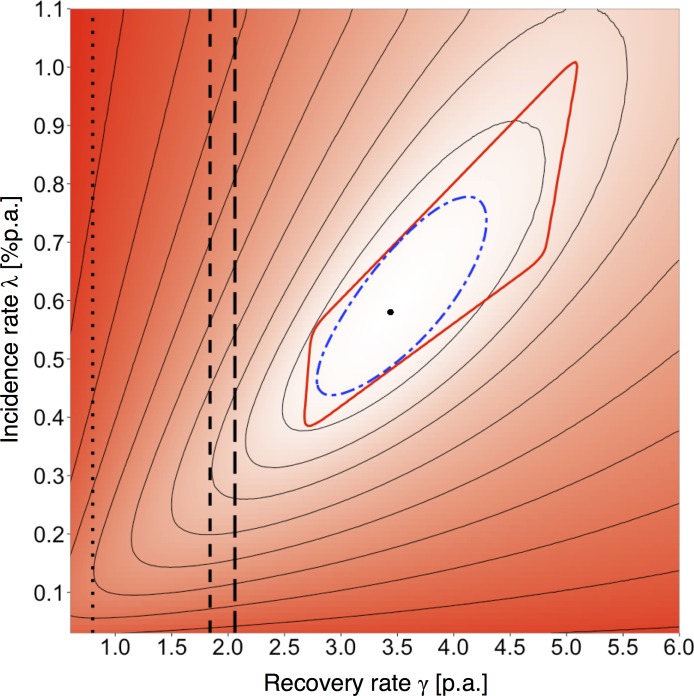
Recovery rate vs. incidence rate, maximum-likelihood estimate and plausible parameter range, Ugandan sex workers. Black dot: maximum-likelihood estimate (MLE) for the recovery rate (*γ* = 3.44p.a.) and the incidence rate (*λ*_*low*_ = 58%p.a.); thin, black contours: equal likelihood; colored areas: white indicates maximum likelihood, red indicated low likelihood; thick, dashed, blue contour: 95% CI of the MLE; thick, red contour: parameter area where the model is consistent with the empirical data (*α*<0.05); vertical lines: estimates from London-student data; dotted: maximum-likelihood estimate (*γ* = 0.80p.a.); short-dashed: highest plausible estimate (*γ*_*max*_ = 1.84p.a.); long-dashed: highest plausible with stable partnership assumption (*γ*_*stable*,*max*_ = 2.06p.a.).

Fig 6 shows the results of a model fit, where we aimed at producing the best fit to the initial prevalence and the apparent clearance as in Fig 1B. The best-fitting parameter combination is *γ* = 3.44p.a. (95% CI: 2.78–4.29); *λ* = 58%p.a. (95% CI: 44%-78%). Neither the confidence intervals, nor the plausible parameter ranges for the London-student (*γ*_*max*_ = 1.84p.a.) and the Ugandan sex-worker (*γ*_*min*_ = 2.67p.a.) models overlap, suggesting that additional factors need to be incorporated in the model to explain both datasets with one common recovery rate.

**Fig 7:**
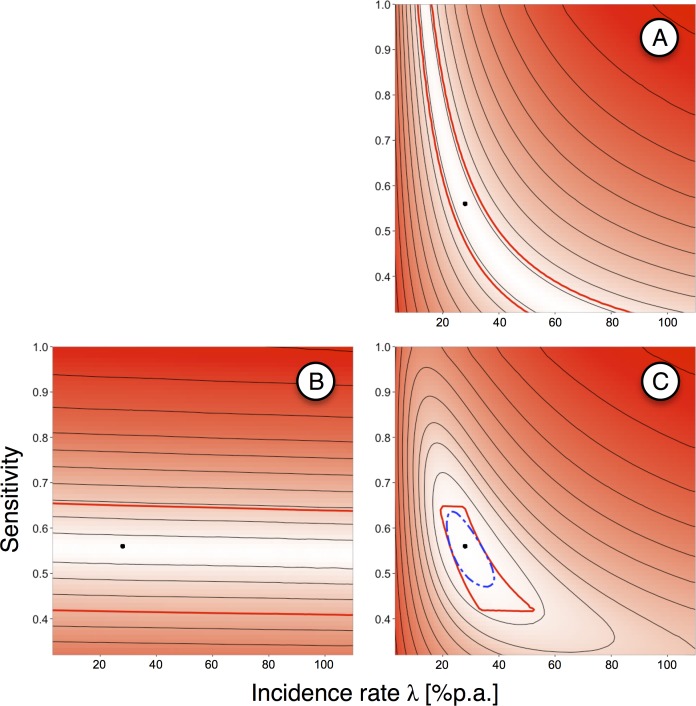
Incidence rate vs. sensitivity, maximum-likelihood estimate and plausible parameter range, Ugandan sex workers. (A) Initial prevalence. (B) continuously *M*. *genitalium*-positive participants over time. (C) Both data items combined. Thick, black dot: maximum-likelihood estimate (MLE) for the incidence rate (*λ*_*low*_ = 28%p.a.) and sensitivity (56%) with recovery rate fixed at *γ* = 0.80p.a. (from London-student data); thin, black contours: equal likelihood; colored areas: white indicates maximum likelihood, red indicated low likelihood; thick, dashed, blue contour: 95% CI of the MLE; thick, red contour: parameter areas where the model is consistent with the empirical data (*α*<0.05).

**Fig 8:**
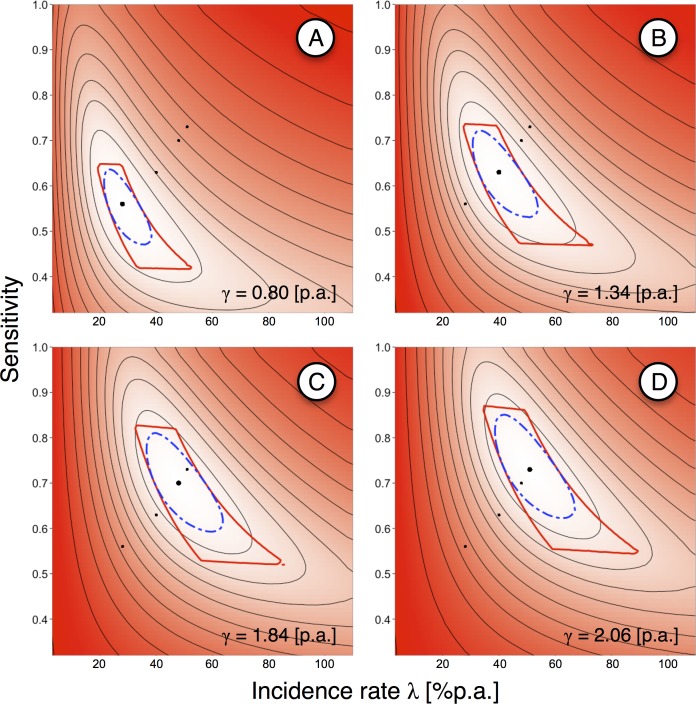
Incidence rate vs. sensitivity, maximum-likelihood estimates and plausible parameter ranges for different recovery rates, Ugandan sex workers. Recovery rates, *γ*, all from London-student data. (A) maximum-likelihood estimate, *γ* = 0.80p.a. (B) upper bound of 95% confidence interval, *γ*_*UCL*_ = *γ*_*stable*_ = 1.34p.a. (C) highest possible estimate, *γ*_*max*_ = 1.84p.a. (D) highest possible estimate when assumed stable partnerships were excluded, *γ*_*stable*,*max*_ = 2.06p.a. Thick, black dots: MLEs for incidence rate and sensitivity with fixed recovery rates; thin, black dots: maximum-likelihood estimates (MLEs) for the other recovery rates; thin, black contours: equal likelihood; colored areas: white indicates maximum likelihood, red indicated low likelihood; thick, dashed, blue contour: 95% CI of the MLE; thick, red contour: parameter areas where the model is consistent with the empirical data (*α*<0.05).

Since one explanation for the apparent differences between the studies in clearing *M*. *genitalium* might be lower testing sensitivity in the Vandepitte et al. study [21], due to different swabbing sites and sample handling, we varied the sensitivity to see if this could ‘reconcile’ the observed patterns (cf. Fig 7). Fig 8 shows sensitivity and incidence estimates for various recovery rates that were derived from the London-student model. For *γ* = 0.80p.a., the best fitting parameter combination was *λ* = 28%p.a. (95% CI: 22%-38%) with a sensitivity of 56% (95% CI: 47%-63%). The highest relative sensitivity that is consistent with the data is 64%. For *γ*_*stable*,*max*_ = 2.06p.a. (i.e., the highest plausible recovery rate in the scenario where all seemingly-persistent infections in the low-risk student group were due to reinfection within a stable partnership), the MLE for the sensitivity is 73% (95% CI: 63%-85%), and the highest plausible sensitivity is 86%.

Vandepitte et al. [21] report that “39% of the women who cleared the infection re-gained positive samples again within 3 to 6 months. Some of these recurrent infections could have been persistent infections after a previous false-negative result”; this reported percentage is consistent with our estimate of that study’s sensitivity, and with *γ* = 0.80p.a., the clearance rate which best explains information from both cohort studies. Higher clearance rates combined with higher sensitivities resulted in substantially lower percentages of participants expected showing this pattern, and they were significantly different from the observed figure and, hence, inconsistent with the empirical data.

## Discussion

We aimed to improve estimates of the recovery and incidence rates of *M*. *genitalium* from cohort studies. Whilst incidence of infection and rates of antibiotic treatment are dependent upon context and patient behavior, and, hence, may vary markedly between studies, rates of natural clearance are likely to be less variable. Estimation of the clearance rate requires adjusting for other factors that are particular to each study setting.

### Factors which impact on clearance rate estimates

There are several factors that might have affected our estimates.

#### Study protocols

Oakeshott et al. [20] used self-taken vaginal swabs and Vandepitte et al. [21] used staff-taken endocervical swabs. Previous studies suggest that vaginal swab specimens have a higher relative sensitivity than endocervical swab specimens [25,26], and that self-obtained specimens appear to be of similar quality as specimens collected by trained staff [25].

Furthermore, Oakeshott et al. [20] stored samples at lower temperatures than Vandepitte et al. [21]: -80°C compared with initial storage at 4°C (up to 12 hours) followed by -20°C for more than two years before they were tested for *M*. *genitalium*. Others have reported that DNA degradation might be slower at -80°C than -20°C [27], affecting test sensitivity.

Also, other factors, such as the transport media, DNA extraction methods, and PCR test kits might have influenced clearance rate estimates. A thorough assessment as to how exactly these differences might have had an impact on test sensitivity cannot be provided at this time, as they have not been studied or findings have not been published, so far.

In light of the discussed protocol differences, we would expect more false-negative test results in the Ugandan study, which would bias parameter estimates towards shorter infection periods because individuals were no longer considered in the published data after their first negative test result. This is corroborated by the agreement of our model results with the frequency of positive-negative-positive results for consecutive time-points, as reported by Vandepitte et al. [21].

A consequence of low relative sensitivities in the Ugandan study is also a higher prevalence than the crude estimate of 14.4%. The highest relative sensitivity estimate compatible with the data is 64%, and the maximum-likelihood estimate is 56%; using these estimates increases the prevalence estimate to 22.5% and 25.7%, respectively. These estimates are consistent with *M*. *genitalium* prevalence estimates for female sex worker populations in developing countries, which were up to 33.5% in Honduras [28] and 26.3% in Ghana and Benin [29].

#### Unintentional treatment

Neither cohort study provided treatment specifically for *M*. *genitalium*. However, individuals with symptomatic infection might have been treated presumptively, and those who were co-infected with another bacterial STI, particularly *C*. *trachomatis* or *N*. *gonorrhoeae*, were given treatment for the co-infection, which might have cured them of *M*. *genitalium*.

Vandepitte et al. reported treating participants with antibiotics, including doxycycline, ciprofloxacin, ceftriaxone, and metronidazole [21]. Doxycycline has a reported cure rate for *M*. *genitalium* of 17%-94% [30]. A wide range of antibiotics are effective or partly effective against at least some *M*. *genitalium* strains [31,32]. Therefore, treatment for non-STI infections might also have unintentionally cleared *M*. *genitalium*. However, Vandepitte et al. did not detect a higher clearance rate in co-infected participants and reported that they did not detect an association between clearance of *M*. *genitalium* and receipt of antibiotics [21].

Although any effect may have been marginal, we expect that unintentional treatment of *M*. *genitalium* would have been more frequent in the Ugandan study, since there was frequent STI testing and treatment [22]. Furthermore, STI co-infection was more common among Ugandan sex workers than London students, particularly for *N*. *gonorrhoeae*, which occurred in 20.9% of *M*. *genitalium*-positive samples from Ugandan sex workers, whilst no co-infection was detected in the London students. In the London study at baseline 10% of *M*. *genitalium* infections were co-infected with *C*. *trachomatis* compared to 12% in the Ugandan study. Due to the difference in *N*. *gonorrhoeae* co-infection, any effect of unintentional treatment of *M*. *genitalium* will have likely been smaller in the London study.

#### Sexual behavior

For *C*. *trachomatis*, it is thought that subclinical infections can be transmitted back and forth between partners in a steady sexual relationship without being detected and treated [33,34]. Since *M*. *genitalium* infections are frequently asymptomatic [20,35], repeated reinfection within stable partnerships (potentially with several cycles of recovery and reinfection) might be an important cause of apparently-persistent infection within an individual when the interval between testing is relatively long, as in the London study. (The authors reported that genotyping suggested patients testing positive at baseline and follow-up had persistent infections, but these results are also consistent with recovery and reinfection within stable partnerships.) This would bias our estimates towards lower recovery rates (i.e., longer infection duration) and lower incidence estimates.

#### Biological differences

The London and Uganda cohorts will have differed in their ethnic composition, with the former being more ethnically diverse [20]. People of different ethnicities might have genetic or other biological differences affecting effectiveness of drug treatments [36] or susceptibility to certain infections [37]. Women of African descent might differ from white women in their immune response to bacterial STIs related to PID [38,39]. Finally, the strains of *M*. *genitalium* in the two populations might have had different phenotypes. However, we have no direct evidence for biological differences and the results of our analyses suggest that any such differences are unlikely to have a substantial impact on the two studies.

Parts of the Uganda cohort were infected with HIV, but Vandepitte et al. [21] found no association between clearance rates of *M*. *genitalium* and HIV status *per se*. Only those HIV-positive participants with a very low CD4 count (<350/ml) had a slower clearance rate than HIV-negatives. With only 16 such individuals in the cohort, modeling this group explicitly will have had little impact on our estimates.

### Interpretation of results

Infection duration in the London-student data appeared to be long (mean duration 15 months), whereas in the Ugandan sex-worker cohort more than half of the initially *M*. *genitalium*-positive participants appeared to have cleared the infection after three months (Fig 3).

We tested the importance of incomplete information by comparing the results of scenarios using different assumptions. Uncertainty in the timing of follow-up testing in the London study makes relatively little difference to estimates, whilst scenarios regarding reinfection result in potentially substantial differences. We identified partnership duration as well as the temporal gap until reinfection within a partnership occurs as critical factors affecting the recovery rate estimate; both are unknown. The basic model for the London data (which ignores partnership duration) might have overestimated the infection duration, but the corresponding MLE also explained recurrent infection in the Ugandan sex workers best. The Ugandan data most likely suggests too-short durations of (untreated) infection because of unintentional treatment of *M*. *genitalium* in co-infected individuals, and probably reduced test sensitivity due to long-term sample storage at -20°C (and, potentially, the choice of transport media or DNA extraction method) and the use of endocervical instead of vaginal swabs. Possibly–although Vandepitte et al. did not detect an effect–unintentional treatment of *M*. *genitalium* in co-infected individuals might have played a minor role.

Incorporating the effects of (i) reinfection within stable partnerships in the London students and (ii) expected lower test sensitivity in the Ugandan sex-worker study in the model result in a common recovery rate to be plausible. Including all available information, the ‘true’ clearance rate appears to be close to the best estimate for the London-student data. Interestingly, both the London-student and the Ugandan sex-worker data are inconsistent with the findings of Tosh et al. [40], who reported that among adolescent women in the USA “31.3% of untreated *M*. *genitalium* cases had infection lasting over 8 weeks”, suggesting a mean duration of <2 months–i.e., a recovery rate >6p.a., which is outside the plausible ranges of both the London-student and the Ugandan sex-worker model. Long-term storage (up to 2 years, although at -70°C) and treatment of co-infections (15/52 *M*. *genitalium*-positive women tested positive for *C*. *trachomatis* and were treated [40]) might have contributed to this high apparent recovery rate (drug resistance in *M*. *genitalium* may have been lower at the time of the study enrollment, 1999–2006, than more recently [7]), but the difference from the other two studies is not fully understood. An in vitro study also suggests that infection may be prolonged [41].

### Future research needs

*M*. *genitalium* is causing increasing concern, particularly regarding if and how it should be managed at the population level, including whether there should be screening or targeted testing [7,9,13,16,17]. Robust parameter estimates are needed for improved understanding of *M*. *genitalium* epidemiology, to inform public health policy, including assessment of cost-effectiveness of intervention strategies.

To better understand the epidemiology of *M*. *genitalium*, we need robust knowledge about fundamental natural-history parameters [17,30,42], including the duration of untreated infection [10], for reliably identifying groups of individuals that are more likely to contract new infections and/or that are more likely to transmit infection to others [43]. (In Britain, 66% of individuals aged 16–44 years who were infected with Chlamydia had not been tested in the last year [44], making the duration of untreated infection an important determinant of transmission.) There is still considerable uncertainty in rates of sequelae due to *C*. *trachomatis* [18], contributing to continuing uncertainty in cost-effectiveness of screening for that infection [16,30]; we suggest that corresponding knowledge gaps for *M*. *genitalium* should be addressed before decision are taken.

Future studies would ideally have larger numbers of initially *M*. *genitalium*-positive participants, followed-up sufficiently frequently to detect changes on timescales of a few weeks [40]. Specimens should be collected from the vagina and from the cervix, if possible, as this results in higher sensitivity [25,26].

Further, ideally, all factors mentioned in the previous section would be controlled for. Collecting more detailed information on sexual behavior and antibiotic use (e.g., Walker et al. [10]) would greatly enhance the value of cohort studies. As we have shown, to estimate the duration of infection, it is important to have information on the incidence (which can lead to recurrent infection) at the individual level, as it is highly heterogeneous: persons in a stable sexual partnership with an infected partner experience a high incidence, typically *greater* than persons who have frequent casual partnerships (but no stable partner), whilst those in an uninfected mutually monogamous partnership have no infection risk. Estimating natural clearance rates requires accounting for antibiotic treatment, including unintentional treatment (e.g., for coinfections).

Finally, as *M*. *genitalium* is a sexually-transmitted infection which infected women typically acquire from men, it is important to better understand its natural history in males; further studies are required.
